# Best environmental predictors of breeding phenology differ with elevation in a common woodland bird species

**DOI:** 10.1002/ece3.6684

**Published:** 2020-08-17

**Authors:** Marjorie Bison, Nigel G. Yoccoz, Bradley Carlson, Geoffrey Klein, Idaline Laigle, Colin Van Reeth, Daphné Asse, Anne Delestrade

**Affiliations:** ^1^ Centre de Recherches sur les Ecosystèmes d’Altitude (CREA Mont‐Blanc) Observatoire du Mont‐Blanc Chamonix France; ^2^ Department of Arctic and Marine Biology UiT The Arctic University of Norway Tromsø Norway; ^3^ Institute of Geography University of Neuchatel Neuchatel Switzerland; ^4^ Centre d’Ecologie Fonctionnelle et Evolutive UMR 5175 CNRS‐Université de Montpellier – Université Paul‐Valéry Montpellier – EPHE Montpellier France

**Keywords:** budburst, climate change, coal tit, elevation, French Alps, laying date, mountain, snow melt‐out

## Abstract

Temperatures in mountain areas are increasing at a higher rate than the Northern Hemisphere land average, but how fauna may respond, in particular in terms of phenology, remains poorly understood. The aim of this study was to assess how elevation could modify the relationships between climate variability (air temperature and snow melt‐out date), the timing of plant phenology and egg‐laying date of the coal tit (*Periparus ater*). We collected 9 years (2011–2019) of data on egg‐laying date, spring air temperature, snow melt‐out date, and larch budburst date at two elevations (~1,300 m and ~1,900 m asl) on a slope located in the Mont‐Blanc Massif in the French Alps. We found that at low elevation, larch budburst date had a direct influence on egg‐laying date, while at high‐altitude snow melt‐out date was the limiting factor. At both elevations, air temperature had a similar effect on egg‐laying date, but was a poorer predictor than larch budburst or snowmelt date. Our results shed light on proximate drivers of breeding phenology responses to interannual climate variability in mountain areas and suggest that factors directly influencing species phenology vary at different elevations. Predicting the future responses of species in a climate change context will require testing the transferability of models and accounting for nonstationary relationships between environmental predictors and the timing of phenological events.

## Graphical abstract

Our study focuses on how the environmental predictors (air temperature, snow melt‐out date, and plant phenology) of breeding phenology (egg‐laying date) differ with elevation in a common woodland bird species, the coal tit.

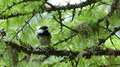

## 1 INTRODUCTION

Climate change is causing large shifts in phenology, that is, the timing of seasonal events, at broad spatial scales and across a wide range of taxa (Dunn, 2004; Menzel et al., 2006; Parmesan & Yohe, 2003; Primack & Gallinat, 2016; Visser, Holleman, & Gienapp, 2006). Variations in the amplitude or direction of such shifts across species (Parmesan, 2007; Thackeray et al., 2016) may lead to mismatch, disrupting species interactions (Burgess et al., 2018; Carter, Saenz, & Rudolf, 2018; Kharouba et al., 2018; Rudolf, 2019) and impacting population dynamics and ecosystem functioning (Both, Van Asch, Bijlsma, Van Den Burg, & Visser, 2009; Miller‐Rushing, Høye, Inouye, & Post, 2010; Thackeray et al., 2010; Visser & Gienapp, 2019; Visser, Noordwijk, Tinbergen, & Lessells, 1998). Breeding phenology is often decisive for the reproductive success of many vertebrate species, as birth or hatching dates have to coincide with resource emergence which itself depends on plant phenology. As air temperature at the beginning of spring increases, species tend to give birth or lay eggs earlier in the season (Cresswell & McCleery, 2003; Dunn, 2004) to maintain synchronization between hatching date and peak in food abundance (Cole, Long, Zelazowski, Szulkin, & Sheldon, 2015; Crick, Dudley, Glue, & Thomson, 1997; Visser et al., 2006) Changes in species phenology may therefore lead to cascading effects in the community and negative consequences on species reproductive success (Dunn, 2004).

Our understanding of animal reproductive phenology in temperate mountain ecosystems is poor (Inouye & Wielgolaski, 2013) due to rough topography and strong seasonality, which render fieldwork difficult. Elevation influences phenological events mainly through a temperature decrease of about 0.6°C/100 m (Dumas, 2013; Körner, 2007; Rolland, 2003). However, in high‐altitude (or high‐latitude) ecosystems, air temperature is not the only forcing climatic factor influencing phenology. Duration and depth of snow cover vary with elevation and topography, and are important parameters influencing mountain species phenology. For example, snow melt‐out date determines the onset of the growing season for alpine plants (Kudo & Hirao, 2006; Vitasse et al., 2017; Wipf, 2010; Wipf, Stoeckli, & Bebi, 2009). The few studies that explored the effects of snow on bird breeding phenology in high elevation ecosystems (see Hendricks, 2003; Morton, 1978; Pereyra, 2011) showed that in North America, breeding is delayed in years of deep snowpack and late snow melt‐out date. Similarly, in the Arctic, it is well established that snow, through depth and melting date, influences the timing of nest initiation of ground‐nesting birds (Dickey, Gauthier, & Cadieux, 2008; Green, Greenwood, & Lloyd, 1977; Hildén, 1965; Liebezeit, Gurney, Budde, Zack, & Ward, 2014; Meltofte, Høye, Schmidt, & Forchhammer, 2007). Recently, Schmidt, Reneerkens, Christensen, Olsen, and Roslin (2019) documented how late snow melt‐out led to catastrophic breeding failure for both plants and animals in the high Arctic.

Few studies, however, have investigated animal phenology along elevation gradients in the same region (but see Nufio, McGuire, Bowers, & Guralnick, 2010 for grasshoppers, Saracco, Siegel, Helton, Stock, & DeSante, 2019 for bird species). Hence, assessing links between changes in climate, or in this case, interannual variability in climate conditions, changes in plant phenology, and changes in bird breeding phenology at different elevations are necessary in order to understand and predict elevation‐dependent phenological responses to climate. Variable importance of climate drivers along elevation gradients could imply a low transferability (Soininen et al., 2018; Yates et al., 2018) of models developed within a narrow elevation band.

In this study, we aimed to quantify and understand relationships between environmental variables and the breeding phenology of a common mountain forest bird species the coal tit (*Periparus ater*), at different elevations on a mountain slope. Coal tits often nest close to the ground and are therefore expected to be dependent on snow conditions, particularly when snowmelt‐out date is late, that is, at high elevation. First, we tested whether annual measures of local environmental variables (air temperature, snow melt‐out date, and tree phenology) explain interannual variation in the timing of coal tit breeding phenology at two elevations. Second, we investigated how elevation modifies the direct and indirect effects of environmental variables on coal tit breeding phenology. Egg‐laying date and environmental variables (air temperature, snow melt‐out date, and plant phenology) were locally measured every year in the field from 2011 to 2019 at two elevations (~1,300 m and ~1,900 m of elevation) in the Mont‐Blanc Massif located in the French Alps.

## 2 MATERIAL AND METHODS

### 2.1 Study site

The study was carried out in Loriaz (46°1′N, 6°55′E), a mountain located above the town of Vallorcine, located in the Mont‐Blanc Massif, France. Forest occurs from 1,300 m to 1,900 m asl and is mainly composed of Norway spruce (*Picea abies*) and European larch (*Larix decidua*). Common birch (*Betula pendula*), rowan (*Sorbus aucuparia*), common hazel (*Corylus avellana*), and European ash (*Fraxinus excelsior*) are mainly present in the lower part of the forest but are much less abundant than coniferous species.

### 2.2 Survey of the breeding phenology of coal tits—Database 1

**Figure 1:**
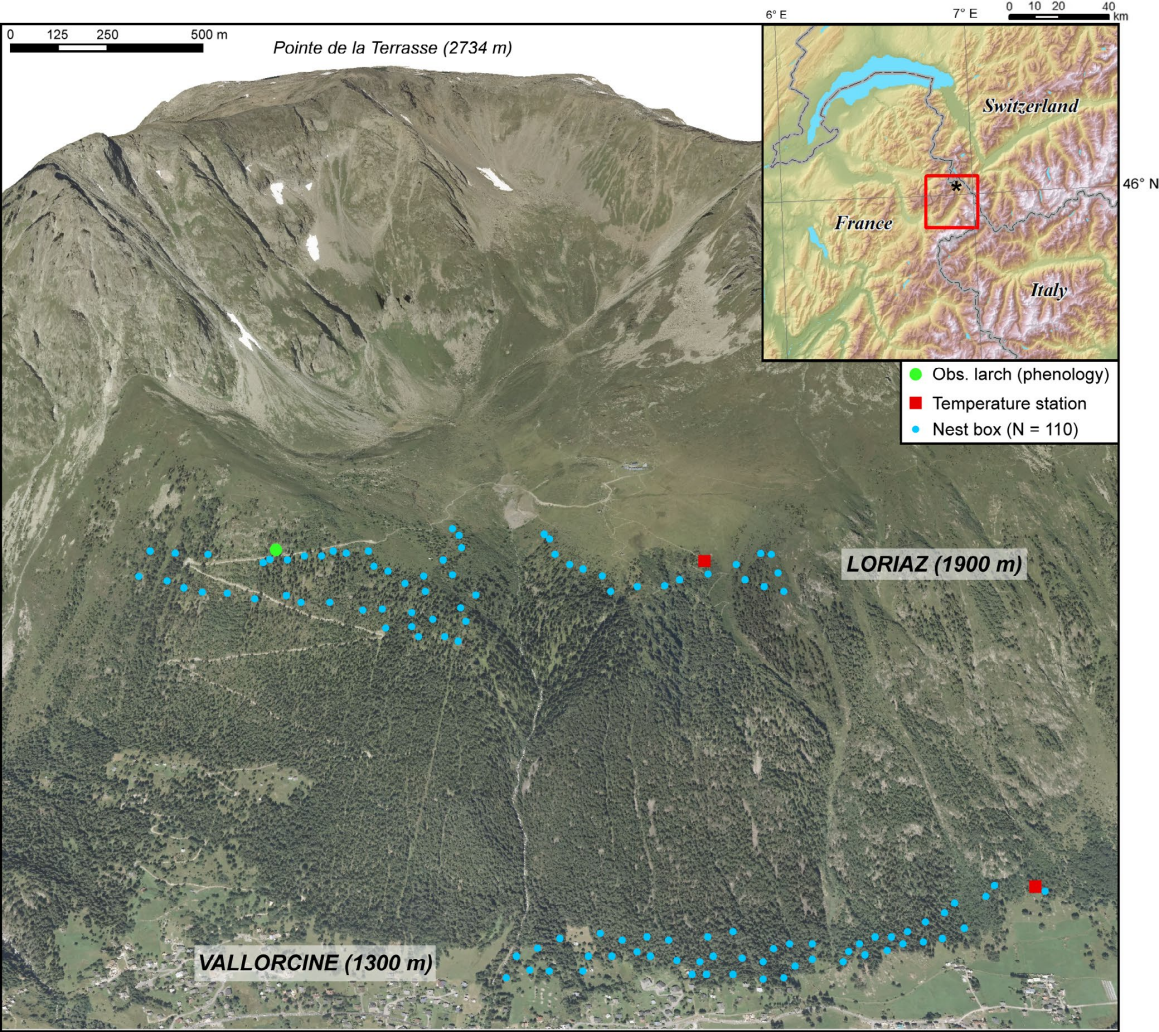
Location map of the nest boxes, climate stations, and larches surveyed. Larches surveyed at the low elevation site are not presented on the map as they are located 5.8 km away to the south, in Montroc

Coal tit (*Periparus ater*) breeding data spanning 9 years (2011–2019) were collected as a part of a long‐term phenology program of the Research Center for Alpine Ecosystems (CREA Mont‐Blanc, Stier et al., 2014, 2016). The coal tit was targeted as a study model as it is a common mountain forest species breeding across wide range of elevations. Coal tits make their nests in a hole in the ground or among tree roots, making them dependent on snow on the ground. One hundred and ten nest boxes were set up along two transects at 1,324 m [1,314 m; 1,334 m] (“low elevation,” LE) and at 1,874 m [1,863 m; 1,885 m] (“high elevation,” HE) (1.4 km and 1.7 km long, respectively; Figure 1).

From April to June, nest boxes were visited weekly. Tits generally lay one egg a day for approximately 8 days (Perrins, 1965 for great tit *Parus major*). Therefore, the laying date (the date when the first egg is laid in a nest) was estimated by counting backwards from the date that clutch was observed. Only the first clutch of the year was used in this study to exclude second breeding events (van Noordwijk, McCleery, & Perrins, 1995). We considered that clutches laid within 30 days of the first clutch of the year were first clutch events (van Noordwijk et al., 1995). From 10% to 45% of nest boxes were occupied in different years and elevations.

### 2.3 Climate data (air temperature and date of snow melt‐out)—Database 2

Climate data were measured by CREA Mont‐Blanc climatic stations located at 1,340 m, 1,915 m, and 1,970 m of elevation on the Loriaz mountain (Mont‐Blanc Massif). The stations located at 1,340 m and 1,915 m of elevation are associated, respectively, with the nest boxes located at low and high elevation (air temperature values at high elevation were missing in 2018 and were interpolated, see ESM 1).

We used the CREA Mont‐Blanc station records (Asse et al., 2018) to calculate mean air temperatures between the 1st of March and the 14th of April. As the air temperatures between the 1st of March and the 25th of April have been shown to significantly influence first egg‐laying date for the great tit (McCleery & Perrins, 1998), we adapted this period to the coal tit by advancing the end of the period to the 14th of April as it is the average of the earliest mean egg‐laying dates at low and high elevation (mean earliest egg‐laying date at LE = 11th of April, HE = 17th of April).

We also tested other time periods (1st of March to 1st of April, 20th of February to 5th of April, 5th of March to 20th of April) to test the robustness of our results. Final results and conclusions are similar as the results obtained with the period 1st of March—14th of April.

We estimated the date of snow melt‐out at each elevation using the temperature sensor located at the ground surface of each station. When snow cover is present, ground surface temperature does not vary and equals 0°C; otherwise, it is positive or negative depending on the air temperature (Gadek & Leszkiewicz, 2010). Date of snow melt‐out corresponds to the last day of snowpack. For each year and each elevation, the timing of snow melt‐out was defined as the first snow‐free day after >40‐day snow‐covered period (~6 weeks) from 1st of September until 31st of August, following Klein, Vitasse, Rixen, Marty, and Rebetez (2016). Given the strong correlation obtained for 2 m height air temperature values between the two highest stations (1,915 m and 1,970 m of elevation), and as there were some missing values for the date of snow melt‐out, we used for the snowmelt‐out date at the high elevation site (ESM 2) the average date of snowmelt‐out by year at the 1,915 m and 1,970 m stations. Snowmelt‐out date at the low elevation site was obtained with the station located at 1,340 m.

### 2.4 Survey of larch budburst—Database 3

As we did not have direct measurement of invertebrate phenology at our study sites, we used larch phenology as a proxy. There is evidence that difference in tree phenological responses may affect bird phenology (Veen et al., 2010), but in our case using larch phenology was the most relevant as it occurred just before egg‐laying while spruce (the other dominant tree species) occurred more than a month later (Pellerin, Delestrade, Mathieu, Rigault, & Yoccoz, 2012, Asse et al., 2018; Bison et al., 2019), spruce buds have a lower frost resistance than larch buds (reviewed in Bigler & Vitasse, 2019). Moreover, there is evidence that invertebrate phenology may be similar between tree taxa (Shutt, Burgess & Phillimore, 2019), at least when considering between‐year variability. The same larches were surveyed every year since 2007. Three mature trees within each site were selected, taller than 7 m and occurring in similar environmental conditions (in terms of soil, slope, aspect, and light). They were observed with binoculars once a week by a researcher of the CREA Mont‐Blanc in spring to record phenological stages (budburst—10% of vegetative buds on a given individual are opened, leafing—10% of the leaves are developed, and flowering—10% of male flowers buds are opened). Because of a lack of budburst data in 2014 and 2015 for larches near the nest boxes at the low elevation site, we used the data of three larches surveyed at another nearby (located 5.8 km away to the south) site located in Montroc (Mont‐Blanc Massif) at ~1,400 m of elevation (Table S1 in ESM 3). Comparisons between the mean budburst date of larches surveyed near the nest boxes for which there is a lack of data in 2014 and 2015 and the mean budburst date of larches surveyed 5.8 km away show similar values and interannual variability (Fig. S1 in ESM 3). At high elevation, three larches were surveyed near the nest box transect at ~1,900 m of elevation (ESM 4). We are aware that the sample size is small at each elevation, but different lines of evidence support that the data provides robust estimate of between‐year variation in budburst dates as (a) the same individuals were surveyed every year, and the differences in budburst dates that we observe among years come mainly from environmental variability and less from interindividual variation (ESM 3), (b) the order of budburst dates among individuals does not vary from one year to another (i.e., the individual that opened its buds the earliest one year always opened its buds the earliest (or the same day as the other trees) the other years).

### 2.5 Data analysis

To estimate temporal trends of mean egg‐laying dates at the two elevations, we used a linear model (egg‐laying date ~ year*elevation), where “*” denotes an interaction between year as a continuous variable and elevation as a categorical variable (high/low) (i.e., different slopes for the year effect). We calculated elevation‐dependent delays for each environmental variable (air temperature, snow melt‐out date, and larch budburst date), that is, the difference between the high and low elevation mean values. To address our first objective, we used linear models to assess the relationships between each environmental variable and coal tit egg‐laying date. We included an interaction term between each variable and elevation to assess elevation‐specific effects. For our second objective, we used structural equation modeling (*SEM*; Shipley, 2002) to estimate the magnitude and significance of hypothesized causal connections between variables (egg‐laying date, air temperature, date of snow melt‐out, and budburst date), and to identify the most important drivers of mean egg‐laying date. In order to detect the elevation‐specific direct and indirect effects of the environmental variables involved in egg‐laying date, we used multigroup analysis applied to *SEM*. This method includes the interaction between the environmental variables and elevation (high/low), and indicates whether the effect of each environmental variable varies with elevation. We first considered a model in which all environmental variables (snowmelt, larch, and temperature) could directly influence egg‐laying date with a model in which only snow and larch budburst directly influenced egg‐laying date using the data at two elevations. Indeed, temperature could directly influence egg‐laying date (Pereyra, 2011) through its influence on gonadal growth (Jones, 1986) and thermoregulation costs of females (Meijer, Nienaber, Langer, & Trillmich, 1999). We then assessed if air temperature had a direct effect on snow melt‐out date and larch budburst date (Asse et al., 2018; Migliavacca et al., 2008), and if snow melt‐out date had an influence on larch budburst date. We accepted the models if their relative goodness of fit *p*‐values (Chi‐square tests) were superior to 0.10 (Grace & Keeley, 2006; Pillar et al., 2013) and selected the model with the lowest AIC, a predictive criterion (Akaike Information Criterion; Claeskens & Hjort, 2008). We then applied multigroup analysis to the selected model (“free” model) by sequentially constraining the coefficients of each path by a single value determined by the entire dataset and refitting the model (“constrained” models). Then, we compared the free and constrained model using a likelihood‐ratio test. If models were significantly different, it implied that the tested path should not be constrained and was differing between sites. Otherwise, it meant that the constrained model was valid and that a single slope value obtained from the entire dataset is suitable for this tested path. Those steps were done for each path. Finally, if several constrained models were valid (*p*‐value > .10, Grace & Keeley, 2006; Pillar et al., 2013), we selected the one with the lowest AIC which indicates that it is the best predictive model. In addition, the path coefficients of the other valid constrained model with the second lowest AIC were compared with the selected one to assess the robustness of the results.

Statistical analyses were carried out using R3.6.1 (R Core Team, 2018), and the “lavaan” package (Rosseel, 2012) was used for *SEM* analyses.

## 3 RESULTS

**Figure 2:**
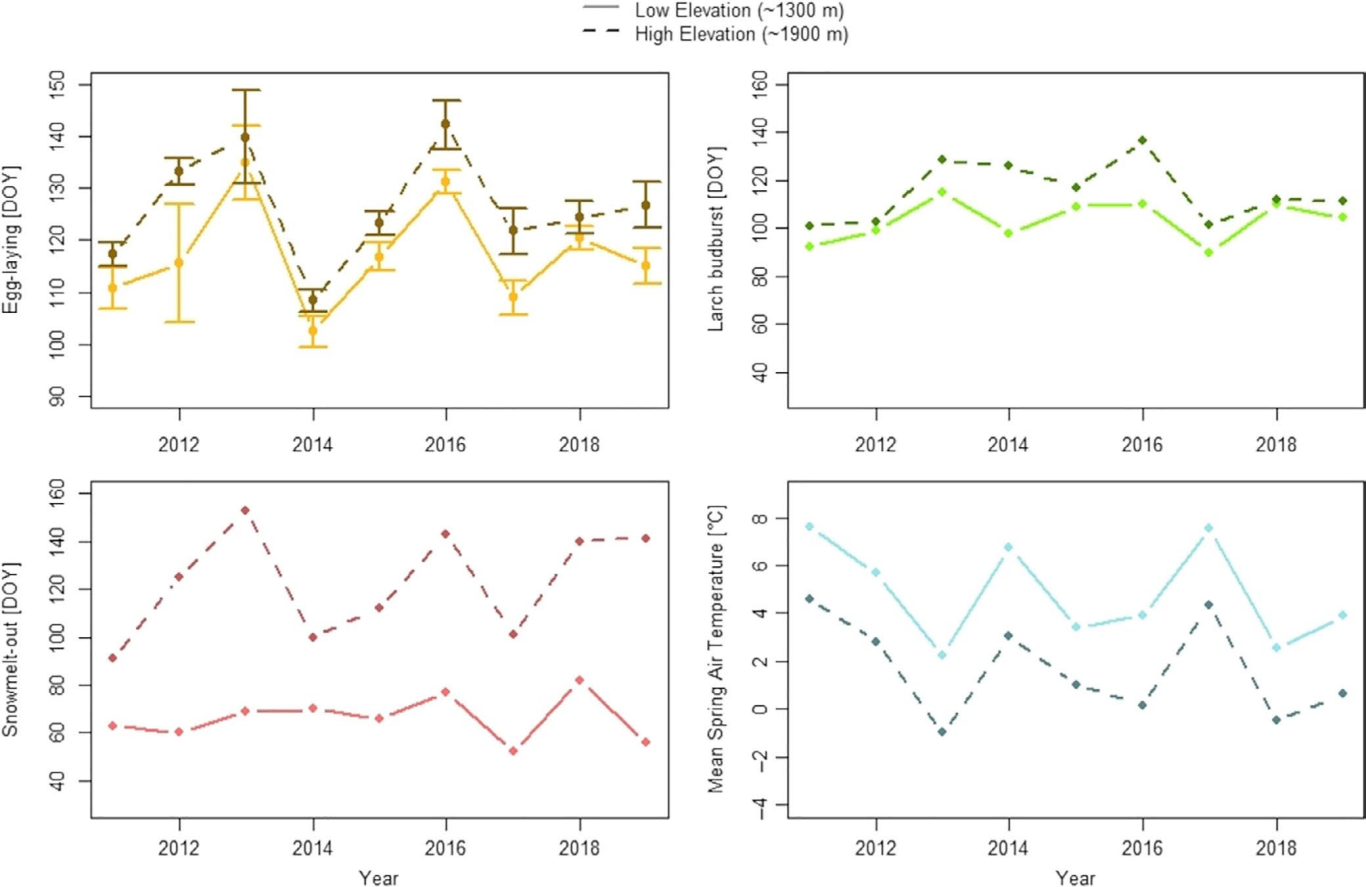
Egg‐laying date, mean larch budburst date, snow melt‐out date, and mean spring temperature variation between 2011 and 2019. 95% confidence intervals are shown for egg‐laying date

Strong interannual variations were observed for egg‐laying date, larch budburst date, snow melt‐out date, and mean air temperature, with events always occurring later at high elevation (Figure 2, Table 1). Year as a continuous variable was not a significant predictor of egg‐laying date at both elevations (0.15 ± 1.06 [*SE*] days later per year), so we could not detect any advancement or delay in the coal tit phenology during the 9 years of the study. We found no clear temporal trend for air temperature (−0.27 ± 0.23 [*SE*] °C per year) during the 2011–2019 period. Elevation‐based differences in air temperature between the low and high elevation sites were similar across years and equaled 3.1°C on average (Table 1, ESM 6). Egg‐laying date, snow melt‐out date, and larch budburst date, however, displayed highly variable responses relative to elevation across years (high coefficient of variation, Table 1, ESM 6).

**Table 1:** Elevational delay between the low and high elevation sites values for each environmental variable (see ESM 6 for a figure). Air temperature is expressed in degrees (°C)

	Egg‐laying date	Air temperature	Larch budburst date	Snow melt‐out date
2011	6.6	3.1	8.7	28
2012	17.6	2.8	4.0	65
2013	4.9	3.2	13.7	84
2014	6.0	3.7	28.0	30
2015	6.4	2.4	8.0	46
2016	11.0	3.7	26.0	66
2017	12.8	3.2	11.3	49
2018	3.9	2.7	2.0	58
2019	11.8	3.3	7.0	85
Mean	9.0	3.1	12.1	56.8
Coefficient of variation	50.3	14.0	75.9	36.4

Average egg‐laying date for the 9 years was negatively correlated with air temperature and positively correlated with larch budburst date and snow melt‐out date (Table 2). In addition, larch budburst date was negatively correlated with air temperature, and snow melt‐out date was negatively correlated with air temperature (Table 2). At both elevations, temperature had similar direct effects on tit egg‐laying date (LE: −3.80 ± 1.20 [*SE*] days/°C, HE: −3.32 ± 1.55 [*SE*] days/°C, Table 2) and on larch budburst date (LE: −4.04 ± 0.40 [*SE*] days/°C, HE: −4.15 ± 1.81 [*SE*] days/°C, Table 2).

**Table 2:** Linear regression models between environmental variables and egg‐laying date at low (“LE”) and high (“HE”) elevations (see ESM 5 for plots). Egg‐laying date, larch budburst date, and snow melt‐out date are expressed in DOY (day of the year). Air temperature is expressed in degrees (°C)

Response variable	Predictors	Slope		Std. error		*R* ^2^		*P*‐value	
		LE	HE	LE	HE	LE	HE	LE	HE
Egg‐laying date	Spring air temp.	−3.80	−3.32	1.20	1.55	0.53	0.31	.02	.07
	Larch budburst date	0.97	0.32	0.26	0.29	0.62	0.03	.007	.30
	Snow melt‐out date	0.49	0.38	0.36	0.11	0.1	0.59	.21	.01
Larch budburst date	Spring air temp.	−4.04	−4.15	0.40	1.81	0.93	0.35	<.001	.06
Snow melt‐out date	Spring air temp.	−2.53	−10.11	1.45	1.75	0.20	0.80	.13	<.001

**Figure 3:**
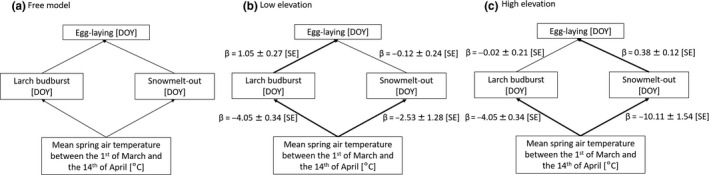
Plausible path analyses for each site (low and high elevation). Slopes (β) are specified with their standard errors. Significant paths are in bold

The free model including the direct effects of snow melt‐out and larch budburst date on egg‐laying date (“M1”) (Figure 3a) was selected over the model including air temperature, snow melt‐out and larch budburst date as direct effect on egg‐laying date (“M2”) (AIC = 385.5 & Goodness of Fit [GOF] *p*‐value = .17 for M1 versus AIC = 387.0 & GOF *p*‐value = .14 for M2). Note also that the latter model, besides being worse in terms of prediction, also led to unstable coefficients because of the correlation between temperature and the other predictors. The inclusion of a path from snow melt‐out to larch budburst in M1 resulted in a model (“M3”) with a p‐value (GOF) of 0.28 indicating an improvement in model fit. However, as the AIC of M3 (385.6) was almost equal to the one of M1 (385.5), we selected the most parsimonious model (i.e., without the direct path between snow melt‐out date and larch budburst date). In addition, this path (“Larch budburst ~ Snow melt‐out date”) was not significant at both elevations, and the effect sizes were low (LE: *β* = 0.15 ± 0.08 [*SE*], HE: *β* = −0.31 ± 0.33 [*SE*]).

The valid model involving the constrained path “Larch budburst ~ Temperature” was selected as it had the lowest AIC (Table 3). Hence, at low elevation, egg‐laying date is directly influenced by larch budburst date: When larch budburst date is one day later, then egg‐laying date is delayed by the same amount (Figure 3b). At high elevation, snow is the main direct driver of egg‐laying date: When the date of snow melt‐out is one day later, then egg‐laying date is 0.38 days later (Figure 3c). The later the snow melts out, the smaller the delay between snow melt‐out date and egg‐laying date. Air temperature had an indirect effect on egg‐laying date either through snowmelt‐out date at high elevation or larch budburst date at low elevation (Figure 3b,c), and based on the *SEM*, egg‐laying date occurs from 3.8 to 3.3 days earlier for every degree (°C) increase, respectively, at low and high elevation. The model including the only direct effect of temperature on egg‐laying date had a higher AIC (385.1) than the model with an indirect effect of the temperature on egg‐laying date through snowmelt‐out date and larch budburst date (Table 3). In addition, it explained 60% and 40% of the variance of egg‐laying date, respectively, at low and high elevation; while the model with the indirect effect of temperature explained 68% and 64% of the variance of egg‐laying date, respectively, at low and high elevation. Hence, the model with an indirect effect of the temperature on egg‐laying date through snowmelt‐out date and larch budburst date better explain variation of egg‐laying date than the model with a single direct effect of temperature. The free model M1, which had the second lowest AIC (Table 3), displayed the same coefficients except for the path “Larch budburst ~ Temperature” which was not constrained in that case (LE: *β* = −4.04 ± 0.35 [*SE*], HE: *β* = −4.15 ± 1.60 [*SE*]).

**Table 3:** AIC, chi‐square test difference, and adjustment quality of the free, entirely constrained and sequentially constrained paths in tested model. Egg‐laying date, larch budburst date, and snow melt‐out date are expressed in DOY (day of the year). Air temperature is expressed in degrees (°C)

	AIC	Chi‐square test difference between the free and constrained model	Goodness of Fit of the model (*p*‐value Chi‐square)
Free model			
Egg‐laying date			
~ Larch budburst			
~ Snow melt‐out			
Snow melt‐out	385.5		.17
~ Air temperature			
Larch budburst			
~ Air temperature			
Entire constrained model	412.3	<0.001	0
Sequentially constrained paths			
Egg‐laying date			
~ Larch budburst	390.6	0.008	.02
Egg‐laying date			
~ Snow melt‐out	386.2	0.10	.10
Snow melt‐out			
~ Air temperature	393.8	0.001	.005
Larch budburst			
~ Air temperature	383.5	0.95	.27

## 4 DISCUSSION

In this study, we aimed to understand direct and indirect relationships between environmental variables and the breeding phenology of a common mountain forest bird species, the coal tit (*Periparus ater*), at two elevations (~1,300 and ~1,900 m). At low elevation, larch budburst date (plant phenology) directly influenced egg‐laying date, and at high elevation, snow melt‐out date was the main predictor. Lower availability of snow‐free ground during years of late snow melt‐out may delay bird breeding phenology of ground‐nesting species such as the coal tit, and delay the availability of ground plant material useful for nest construction (moss and animal hairs for coal tit nests, Pereyra, 2011; Saracco et al., 2019). Alternatively, at low elevation, to maintain synchronization, later plant phenology (larch budburst date) may delay bird breeding phenology due to later insect emergence (Finn & Poff, 2008; Marshall, Cooper, DeCecco, Strazanac, & Butler, 2002). Here, we cannot determine whether it is plant phenology acting as an environmental cue for coal tit individuals (Cole et al., 2015; Hinks et al., 2015; Thomas, Bourgault, Shipley, Perret, & Blondel, 2010) or other factors co‐occurring with budburst, such as insect emergence, that initiate egg‐laying date. Future work should be conducted at a larger spatial scale to better identify how local environment influences bird nesting, as some experimental evidence suggests that plant phenology may not be the main environmental cue for the onset of bird breeding (Schaper, Rueda, Sharp, Dawson, & Visser, 2011; Visser, Silverin, Lambrechts, & Tinbergen, 2002). In addition, a larger sample size of budburst dates, both within species (larch) and across species, could be surveyed to explore the relationship between interindividual and interspecific variation in budburst and breeding phenology. While the relationships found in our study have been independently detected for other bird species (effect of plant phenology at low elevation in tits in Thomas et al., 2010, Hinks et al., 2015, Cole et al., 2015 and Shutt, Cabello, et al., 2019; effect of snow at high elevation in American pipit, Dusky flycatcher and White‐crowned sparrows, respectively, in Hendricks, 2003, Pereyra, 2011 and Morton, 1978) our study shows that elevation determines which predictors are best (Saracco et al., 2019).

In addition, our results concur with other studies on woodland passerines indicating that air temperature has a strong causal effect on egg‐laying dates (Pereyra, 2011; Phillimore, Leech, Pearce‐Higgins, & Hadfield, 2016; Shutt, Burgess, et al., 2019; Shutt, Cabello, et al., 2019; Simmonds, Cole, & Sheldon, 2019; Visser, Holleman, & Caro, 2009). Our study improves the understanding of mechanisms involved in the determining factors of bird breeding phenology by showing that air temperature has a negative indirect effect through plant phenology or snow melt‐out date, depending on the elevation. If air temperature alone would have explained egg‐laying date, elevation‐dependent delay in egg‐laying date would have been constant over the years. Specifically, based on the *SEM*, egg‐laying date occurs from 3.8 to 3.3 days earlier for every degree, respectively, at low and high elevation, which is similar to blue and great tit in UK (−4 days/°C, Burgess et al., 2018). Hence, the effect of temperature on egg‐laying date is similar at both elevations but underlying mechanisms may differ.

While much evidence indicates that European mountain areas experienced spring warming (up to ~0.8°C/decade in April) since 1970 (Klein, Rebetez, Rixen, & Vitasse, 2018, see also Nilsen, Fleig, Tallasken, & Hisdal, 2014 for the period 1979–2009), our study period was not long enough to detect trends in egg‐laying date (Parmesan, 2006), a pattern confirmed by the absence of temporal trend of air temperature. However, the strong interannual climate variability led us to hypothesize high plasticity in the breeding phenology of the coal tit in response to these environmental variations, which was already observed at the individual level for the great tit (Charmantier et al., 2008).

According to the scenario RCP4.5 (scenario of long‐term global emissions of greenhouse gases which stabilizes radiative forcing at 4.5 W/m^2^ in the year 2100), temperature is expected to increase by 2 to 3°C in the Alps by the end of the 21st century, with potentially increased rates of warming at higher elevation (Gobiet et al., 2014; Jacob et al., 2014). Simultaneously, climate models predict that the snow amount and duration will drastically decrease below 1,500–2,000 m of elevation, in particular, due to earlier snow melt‐out in spring (Beniston, 2012; Beniston et al. 2018; Beniston, Keller, Koffi, & Goyette, 2003; Castebrunet, Eckert, Giraud, Durand, & Morin, 2014; Gobiet et al. 2014; Verfaillie et al. 2018). Furthermore, models predict increasing rainfall during the winter months as well as higher frequency of heavy precipitation events, which could contribute to a reduced and faster melting snowpack (Jacob et al. 2014).

Given these climate projections, we expect that bird breeding phenology will advance until the end of the 21st century. At our high elevation site (~1,900 m asl), a thinner winter snowpack and earlier spring snow melt‐out may lead to earlier onset of breeding. However, we hypothesize that under a certain threshold of snow melt‐out date, this climatic factor will not have as much influence compared with current conditions. Hence, we expect that the relationships between variables of the high‐altitude site (i.e., direct snow melt‐out date effect on egg‐laying date) may move toward the relationships of the low altitude site (i.e., direct larch budburst date effect on egg‐laying date). Finally, considering that observed warming is stronger at higher elevations, we expect that the delay in breeding phenology between both elevations might decrease (Vitasse, Signarbieux, & Fu, 2018).

In conclusion, our study demonstrates how the predictors of bird breeding phenology can vary with elevation. Larch budburst date and snow melt‐out date are significant direct predictors of egg‐laying dates of the coal tit, respectively, at low and high elevation, while temperature has a significant indirect effect in both cases. Variable phenological responses according to elevation raise the question of transferability (Soininen et al. 2018; Yates et al. 2018) of models built at a single elevation as the predictors of phenology at one elevation may not be consistent at higher elevations, or relevant in the context of future climate changes. Additional long‐term studies on animal phenology in mountain environments should be conducted in order to better predict how interactions between species will change in the context of climate change in mountain areas, where responses can differ along elevation gradients.

## CONFLICT OF INTEREST

None declared.

## AUTHOR CONTRIBUTION

**Marjorie Bison:** Conceptualization (equal); Data curation (equal); Formal analysis (equal); Methodology (equal); Validation (equal); Visualization (equal); Writing‐original draft (lead); Writing‐review & editing (equal). **Nigel Yoccoz:** Conceptualization (equal); Formal analysis (equal); Methodology (equal); Supervision (equal); Validation (equal); Writing‐original draft (equal); Writing‐review & editing (equal). **Bradley Carlson:** Conceptualization (equal); Data curation (equal); Methodology (equal); Validation (equal); Visualization (equal); Writing‐original draft (equal); Writing‐review & editing (equal). **Geoffrey Klein:** Conceptualization (equal); Data curation (equal); Methodology (equal); Validation (equal); Visualization (equal); Writing‐original draft (equal); Writing‐review & editing (equal). **Idaline Laigle:** Data curation (equal); Methodology (equal); Validation (equal); Visualization (equal); Writing‐original draft (equal); Writing‐review & editing (equal). **Colin Van Reeth:** Data curation (equal); Methodology (equal); Validation (equal); Visualization (equal); Writing‐original draft (equal); Writing‐review & editing (equal). **Daphné Asse:** Data curation (equal); Methodology (equal); Validation (equal); Visualization (equal); Writing‐original draft (equal); Writing‐review & editing (equal). **Anne Delestrade:** Conceptualization (equal); Data curation (equal); Funding acquisition (equal); Methodology (equal); Project administration (equal); Supervision (equal); Validation (equal); Visualization (equal); Writing‐original draft (equal); Writing‐review & editing (equal)..

## Supporting information

Supinfo

## Data Availability

Data are deposited on Dryad and accessible here: https://datadryad.org/stash/share/X2rab‐pvvDzXpdSGJzPmMKXW8RiCCFEt_tWF4ty9Xrk.

## References

[ece36684-bib-0001] Asse, D. , Chuine, I. , Vitasse, Y. , Yoccoz, N. G. , Delpierre, N. , Badeau, V. , … Randin, C. F. (2018). Warmer winters reduce the advance of tree spring phenology induced by warmer springs in the Alps. Agricultural and Forest Meteorology, 252, 220–230. 10.1016/j.agrformet.2018.01.030

[ece36684-bib-0002] Beniston, M. (2012). Is snow in the Alps receding or disappearing? Wiley Interdisciplinary Reviews: Climate Change, 3(4), 349–358. 10.1002/wcc.179

[ece36684-bib-0003] Beniston, M. , Farinotti, D. , Stoffel, M. , Andreassen, L. M. , Coppola, E. , Eckert, N. , … Vincent, C. (2018). The European mountain cryosphere: A review of its current state, trends and future challenges. Cryosphere, 12(2), 759–794. 10.5194/tc-12-759-2018

[ece36684-bib-0004] Beniston, M. , Keller, F. , Koffi, B. , & Goyette, S. (2003). Estimates of snow accumulation and volume in the Swiss Alps under changing climatic conditions. Theoretical and Applied Climatology, 76(3–4), 125–140. 10.1007/s00704-003-0016-5

[ece36684-bib-0005] Bigler, C. , & Vitasse, Y. (2019). Daily maximum temperatures induce lagged effects on leaf unfolding in temperate woody species across large elevational gradients. Frontiers in Plant Science, 10, 398 10.3389/fpls.2019.00398 30984231 PMC6447654

[ece36684-bib-0006] Bison, M. , Yoccoz, N. G. , Carlson, B. , & Delestrade, A. (2019). Comparison of budburst phenology trends and precision among participants in a citizen science program. International Journal of Biometeorology, 63(1), 61–72. 10.1007/s00484-018-1636-x 30382351

[ece36684-bib-0007] Both, C. , Van Asch, M. , Bijlsma, R. G. , Van Den Burg, A. B. , & Visser, M. E. (2009). Climate change and unequal phenological changes across four trophic levels: Constraints or adaptations? Journal of Animal Ecology, 78(1), 73–83. 10.1111/j.1365-2656.2008.01458.x 18771506

[ece36684-bib-0008] Burgess, M. D. , Smith, K. W. , Evans, K. L. , Leech, D. , Pearce‐Higgins, J. W. , Branston, C. J. , … Phillimore, A. B. (2018). Tritrophic phenological match–mismatch in space and time. Nature Ecology & Evolution, 2(6), 970–975. 10.1038/s41559-018-0543-1 29686235

[ece36684-bib-0009] Carter, S. K. , Saenz, D. , & Rudolf, V. H. (2018). Shifts in phenological distributions reshape interaction potential in natural communities. Ecology Letters, 21(8), 1143–1151. 10.1111/ele.13081 29927047

[ece36684-bib-0010] Castebrunet, H. , Eckert, N. , Giraud, G. , Durand, Y. , & Morin, S. (2014). Projected changes of snow conditions and avalanche activity in a warming climate: A case study in the French Alps over the 2020–2050 and 2700–2100 periods. The Cryosphere, 8, 581–640. 10.5194/tc-8-1673-2014

[ece36684-bib-0011] Charmantier, A. , McCleery, R. H. , Cole, L. R. , Perrins, C. , Kruuk, L. E. B. , & Sheldon, B. C. (2008). Adaptive phenotypic plasticity in response to climate change in a wild bird population. Science, 320(5877), 800–803. 10.1126/science.1157174 18467590

[ece36684-bib-0012] Claeskens, G. , & Hjort, N. L. (2008). Model selection and model averaging. Cambridge: Cambridge University Press.

[ece36684-bib-0013] Cole, E. F. , Long, P. R. , Zelazowski, P. , Szulkin, M. , & Sheldon, B. C. (2015). Predicting bird phenology from space: Satellite‐derived vegetation green‐up signal uncovers spatial variation in phenological synchrony between birds and their environment. Ecology and Evolution, 5(21), 5057–5074. 10.1002/ece3.1745 26640682 PMC4662320

[ece36684-bib-0014] Cresswell, W. , & McCleery, R. (2003). How great tits maintain synchronization of their hatch date with food supply in response to long‐term variability in temperature. Journal of Animal Ecology, 72(2), 356–366. 10.1046/j.1365-2656.2003.00701.x

[ece36684-bib-0015] Crick, H. Q. , Dudley, C. , Glue, D. E. , & Thomson, D. L. (1997). UK birds are laying eggs earlier. Nature, 388(6642), 526 10.1038/41453

[ece36684-bib-0016] Dickey, M.‐H. , Gauthier, G. , & Cadieux, M.‐C. (2008). Climatic effects on the breeding phenology and reproductive success of an arctic‐nesting goose species. Global Change Biology, 14(9), 1973–1985. 10.1111/j.1365-2486.2008.01622.x

[ece36684-bib-0017] Dumas, M. D. (2013). Changes in temperature and temperature gradients in the French Northern Alps during the last century. Theoretical and Applied Climatology, 111(1–2), 223–233. 10.1007/s00704-012-0659-1

[ece36684-bib-0018] Dunn, P. (2004). Breeding dates and reproductive performance. Advances in Ecological Research, 35, 69–87. 10.1016/S0065-2504(04)35004-X

[ece36684-bib-0019] Finn, D. S. , & Poff, N. L. (2008). Emergence and flight activity of alpine stream insects in two years with contrasting winter snowpack. Arctic, Antarctic, and Alpine Research, 40(4), 638–646. 10.1657/1523-0430(07-072)[FINN]2.0.CO;2

[ece36684-bib-0020] Gadek, B. , & Leszkiewicz, J. (2010). Influence of snow cover on ground surface temperature in the zone of sporadic permafrost, Tatra Mountains, Poland and Slovakia. Cold Regions Science and Technology, 60(3), 205–211. 10.1016/j.coldregions.2009.10.004

[ece36684-bib-0021] Gobiet, A. , Kotlarski, S. , Beniston, M. , Heinrich, G. , Rajczak, J. , & Stoffel, M. (2014). 21st century climate change in the European Alps ‐ a review. Science of the Total Environment, 493, 1138–1151. 10.1016/j.scitotenv.2013.07.050 23953405

[ece36684-bib-0022] Grace, J. B. , & Keeley, J. E. (2006). A structural equation model analysis of postfire plant diversity in California shrublands. Ecological Applications, 16(2), 503–514. 10.1890/1051-0761(2006)016[0503:ASEMAO]2.0.CO;2 16711040

[ece36684-bib-0023] Green, G. , Greenwood, J. , & Lloyd, C. (1977). The influence of snow conditions on the date of breeding of wading birds in north‐east Greenland. Journal of Zoology, 183(3), 311–328. 10.1111/j.1469-7998.1977.tb04190.x

[ece36684-bib-0024] Hendricks, P. (2003). Spring snow conditions, laying date, and clutch size in an alpine population of American pipits. Journal of Field Ornithology, 74(4), 423–430. 10.1648/0273-8570-74.4.423

[ece36684-bib-0025] Hildén, O. (1965). Habitat selection in birds: A review. Annales Zoologici Fennici, 2(1), 53–75.

[ece36684-bib-0026] Hinks, A. E. , Cole, E. F. , Daniels, K. J. , Wilkin, T. A. , Nakagawa, S. , & Sheldon, B. C. (2015). Scale‐dependent phenological synchrony between songbirds and their caterpillar food source. The American Naturalist, 186(1), 84–97. 10.1086/681572 26098341

[ece36684-bib-0027] Inouye, D. W. , & Wielgolaski, F. E. (2013). Phenology at high altitudes. Dordrecht: Springer.

[ece36684-bib-0028] Jacob, D. , Petersen, J. , Eggert, B. , Alias, A. , Christensen, O. B. , Bouwer, L. M. , … Yiou, P. (2014). EURO‐CORDEX: New high‐resolution climate change projections for European impact research. Regional Environmental Change, 14(2), 563–578. 10.1007/s10113-013-0499-2

[ece36684-bib-0029] Jones, L. R. (1986). The effect of photoperiod and temperature on testicular growth in captive black‐billed magpies. The Condor, 88(1), 91–93. 10.2307/1367759

[ece36684-bib-0030] Kharouba, H. M. , Ehrlén, J. , Gelman, A. , Bolmgren, K. , Allen, J. M. , Travers, S. E. , & Wolkovich, E. M. (2018). Global shifts in the phenological synchrony of species interactions over recent decades. Proceedings of the National Academy of Sciences, 115(20), 5211–5216. 10.1073/pnas.1714511115 PMC5960279 29666247

[ece36684-bib-0031] Klein, G. , Rebetez, M. , Rixen, C. , & Vitasse, Y. (2018). Unchanged risk of frost exposure for subalpine and alpine plants after snow melt‐out in Switzerland despite climate warming. International Journal of Biometeorology, 62(9), 1755–1762. 10.1007/s00484-018-1578-3 30003338

[ece36684-bib-0032] Klein, G. , Vitasse, Y. , Rixen, C. , Marty, C. , & Rebetez, M. (2016). Shorter snow cover duration since 1970 in the Swiss Alps due to earlier snow melt‐out more than to later snow onset. Climatic Change, 139(3–4), 637–649. 10.1007/s10584-016-1806-y

[ece36684-bib-0033] Körner, C. (2007). The use of ‘altitude’ in ecological research. Trends in Ecology & Evolution, 22(11), 569–574. 10.1016/j.tree.2007.09.006 17988759

[ece36684-bib-0034] Kudo, G. , & Hirao, A. S. (2006). Habitat‐specific responses in the flowering phenology and seed set of alpine plants to climate variation: Implications for global‐change impacts. Population Ecology, 48(1), 49–58. 10.1007/s10144-005-0242-z

[ece36684-bib-0035] Liebezeit, J. R. , Gurney, K. , Budde, M. , Zack, S. , & Ward, D. (2014). Phenological advancement in arctic bird species: Relative importance of snow melt and ecological factors. Polar Biology, 37(9), 1309–1320. 10.1007/s00300-014-1522-x

[ece36684-bib-0036] Marshall, M. R. , Cooper, R. J. , DeCecco, J. A. , Strazanac, J. , & Butler, L. (2002). Effects of experimentally reduced prey abundance on the breeding ecology of the red‐eyed vireo. Ecological Applications, 12(1), 261–280. 10.1890/1051-0761(2002)012[0261:EOERPA]2.0.CO;2

[ece36684-bib-0037] McCleery, R. , & Perrins, C. (1998). Temperature and egg‐laying trends. Nature, 391(6662), 30 10.1038/34073

[ece36684-bib-0038] Meijer, T. , Nienaber, U. , Langer, U. , & Trillmich, F. (1999). Temperature and timing of egg‐laying of European starlings. The Condor, 101(1), 124–132. 10.2307/1370453

[ece36684-bib-0039] Meltofte, H. , Høye, T. T. , Schmidt, N. M. , & Forchhammer, M. C. (2007). Differences in food abundance cause inter‐annual variation in the breeding phenology of high arctic waders. Polar Biology, 30(5), 601 10.1007/s00300-006-0219-1

[ece36684-bib-0040] Menzel, A. , Sparks, T. H. , Estrella, N. , Koch, E. , Aasa, A. , Ahas, R. , … Zust, A. (2006). European phenological response to climate change matches the warming pattern. Global Change Biology, 12(10), 1969–1976. 10.1111/j.1365-2486.2006.01193.x

[ece36684-bib-0041] Migliavacca, M. , Cremonese, E. , Colombo, R. , Busetto, L. , Galvagno, M. , Ganis, L. , … Morra di Cella, U. (2008). European larch phenology in the Alps: Can we grasp the role of ecological factors by combining field observations and inverse modelling? International Journal of Biometeorology, 52(7), 587–605. 10.1007/s00484-008-0152-9 18437430

[ece36684-bib-0042] Miller‐Rushing, A. J. , Høye, T. T. , Inouye, D. W. , & Post, E. (2010). The effects of phenological mismatches on demography. Philosophical Transactions of the Royal Society of London B: Biological Sciences, 365(1555), 3177–3186. 10.1098/rstb.2010.0148 20819811 PMC2981949

[ece36684-bib-0043] Morton, M. L. (1978). Snow conditions and the onset of breeding in the mountain white‐crowned sparrow. The Condor, 80(3), 285–289. 10.2307/1368037

[ece36684-bib-0044] Nilsen, I. B. , Fleig, A. K. , Tallasken, L. M. , & Hisdal, H. (2014). Recent trends in monthly temperature and precipitation patterns in Europe In Daniell T. M., Van Lanen H. A. J., Demuth S., Laaha G., Servat E., Mahe G., et al. (Eds.), Hydrology in a changing world: Environmental and human dimensions (pp. 132‐137). International Association of Hydrological Sciences.

[ece36684-bib-0045] Nufio, C. R. , McGuire, C. R. , Bowers, M. D. , & Guralnick, R. P. (2010). Grasshopper community response to climatic change: Variation along an elevational gradient. PLoS One, 5(9), e12977 10.1371/journal.pone.0012977 20886093 PMC2944887

[ece36684-bib-0046] Parmesan, C. (2006). Ecological and evolutionary responses to climate change. Annual Review of Ecology, Evolution, and Systematics, 37, 637–669. 10.1146/annurev.ecolsys.37.091305.110100

[ece36684-bib-0047] Parmesan, C. (2007). Influences of species, latitudes and methodologies on estimates of phenological response to global warming. Global Change Biology, 13(9), 1860–1872. 10.1111/j.1365-2486.2007.01404.x

[ece36684-bib-0048] Parmesan, C. , & Yohe, G. (2003). A globally coherent fingerprint of climate change impacts across natural systems. Nature, 421(6918), 37 10.1038/nature01286 12511946

[ece36684-bib-0049] Pellerin, M. , Delestrade, A. , Mathieu, G. , Rigault, O. , & Yoccoz, N. G. (2012). Spring tree phenology in the Alps: Effects of air temperature, altitude and local topography. European Journal of Forest Research, 131(6), 1957–1965. 10.1007/s10342-012-0646-1

[ece36684-bib-0050] Pereyra, M. E. (2011). Effects of snow‐related environmental variation on breeding schedules and productivity of a high‐altitude population of dusky flycatchers (*Empidonax oberholseri*). The Auk, 128(4), 746–758. 10.1525/auk.2011.10144

[ece36684-bib-0051] Perrins, C. M. (1965). Population fluctuations and clutch‐size in the great tit, *Parus major* . The Journal of Animal Ecology, 34, 601–647. 10.2307/2453

[ece36684-bib-0052] Phillimore, A. B. , Leech, D. , Pearce‐Higgins, J. W. , & Hadfield, J. D. (2016). Passerines may be sufficiently plastic to track temperature‐mediated shifts in optimum lay date. Global Change Biology, 22(10), 3259–3272. 10.1111/gcb.13302 27173755

[ece36684-bib-0053] Pillar, V. D. , Blanco, C. C. , Müller, S. C. , Sosinski, E. E. , Joner, F. , & Duarte, L. D. S. (2013). Functional redundancy and stability in plant communities. Journal of Vegetation Science, 24(5), 963–974. 10.1111/jvs.12047

[ece36684-bib-0054] Primack, R. , & Gallinat, A. (2016). Spring budburst in a changing climate. American Scientist, 104, 102–109.

[ece36684-bib-0055] R Core Team (2018). R: A language and environment for statistical computing. Vienne, Austria: R Foundation for Statistical Computing https://www.R‐project.org/

[ece36684-bib-0056] Rolland, C. (2003). Spatial and seasonal variations of air temperature lapse rates in alpine regions. Journal of Climate, 16(7), 1032–1046. 10.1175/1520-0442(2003)016<1032:SASVOA>2.0.CO;2

[ece36684-bib-0057] Rosseel, Y. (2012). lavaan: An R package for structural equation modeling. Journal of Statistical Software, 48(2), 1–36.

[ece36684-bib-0058] Rudolf, V. H. (2019). The role of seasonal timing and phenological shifts for species coexistence. Ecology Letters, 22(8), 1324–1338. 10.1111/ele.13277 31125170

[ece36684-bib-0059] Saracco, J. F. , Siegel, R. B. , Helton, L. , Stock, S. L. , & DeSante, D. F. (2019). Phenology and productivity in a montane bird assemblage: Trends and responses to elevation and climate variation. Global Change Biology, 25(3), 985–996. 10.1111/gcb.14538 30506620

[ece36684-bib-0060] Schaper, S. V. , Rueda, C. , Sharp, P. J. , Dawson, A. , & Visser, M. E. (2011). Spring phenology does not affect timing of reproduction in the great tit (*Parus major*). Journal of Experimental Biology, 214(21), 3664–3671. 10.1242/jeb.059543 21993796

[ece36684-bib-0061] Schmidt, N. M. , Reneerkens, J. , Christensen, J. H. , Olsen, M. , & Roslin, T. (2019). An ecosystem‐wide reproductive failure with more snow in the Arctic. PLoS Biology, 17(10), 10.1371/journal.pbio.3000392 PMC6793841 31613872

[ece36684-bib-0062] Shipley, B. (2002). Cause and correlation in biology: A user’s guide to path analysis, structural equations and causal inference. Cambridge: Cambridge University Press.

[ece36684-bib-0063] Shutt, J. D. , Burgess, M. D. , & Phillimore, A. B. (2019). A spatial perspective on the phenological distribution of the spring woodland caterpillar peak. The American Naturalist, 194(5), E109–E121. 10.1086/705241 31613670

[ece36684-bib-0064] Shutt, J. D. , Cabello, I. B. , Keogan, K. , Leech, D. I. , Samplonius, J. M. , Whittle, L. , … Phillimore, A. B. (2019). The environmental predictors of spatio‐temporal variation in the breeding phenology of a passerine bird. Proceedings of the Royal Society B, 286(1908), 20190952 10.1098/rspb.2019.0952 31409248 PMC6710590

[ece36684-bib-0065] Simmonds, E. G. , Cole, E. F. , & Sheldon, B. C. (2019). Cue identification in phenology: A case study of the predictive performance of current statistical tools. Journal of Animal Ecology, 88, 1428–1440. 10.1111/1365-2656.13038 31162635 PMC8629117

[ece36684-bib-0066] Soininen, E. M. , Henden, J. H. , Ravolainen, V. T. , Yoccoz, N. G. , Bråthen, K. A. , Killengreen, S. T. , & Ims, R. A. (2018). Transferability of biotic interactions: Temporal consistency of arctic plant–rodent relationships is poor. Ecology and Evolution, 8(19), 9697–9711. 10.1002/ece3.4399 30386568 PMC6202721

[ece36684-bib-0067] Stier, A. , Delestrade, A. , Bize, P. , Zahn, S. , Criscuolo, F. , & Massemin, S. (2016). Investigating how telomere dynamics, growth and life history covary along an elevation gradient in two passerine species. Journal of Avian Biology, 47(1), 134–140. 10.1111/jav.00714

[ece36684-bib-0068] Stier, A. , Delestrade, A. , Zahn, S. , Arrivé, M. , Criscuolo, F. , & Massemin‐Challet, S. (2014). Elevation impacts the balance between growth and oxidative stress in coal tits. Oecologia, 175(3), 791–800. 10.1007/s00442-014-2946-2 24805201

[ece36684-bib-0069] Thackeray, S. J. , Henrys, P. A. , Hemming, D. , Bell, J. R. , Botham, M. S. , Burthe, S. , … Wanless, S. (2016). Phenological sensitivity to climate across taxa and trophic levels. Nature, 535(7611), 241–245. 10.1038/nature18608 27362222

[ece36684-bib-0070] Thackeray, S. J. , Sparks, T. H. , Frederiksen, M. , Burthe, S. , Bacon, P. J. , Bell, J. R. , … Wanless, S. (2010). Trophic level asynchrony in rates of phenological change for marine, freshwater and terrestrial environments. Global Change Biology, 16(12), 3304–3313.

[ece36684-bib-0071] Thomas, D. W. , Bourgault, P. , Shipley, B. , Perret, P. , & Blondel, J. (2010). Context‐dependent changes in the weighting of environmental cues that initiate breeding in a temperate passerine, the Corsican blue tit (*Cyanistes caeruleus*). The Auk, 127(1), 129–139. 10.1525/auk.2009.09141

[ece36684-bib-0072] Van Noordwijk, A. J. , McCleery, R. H. , & Perrins, C. M. (1995). Selection for the timing of great tit breeding in relation to caterpillar growth and temperature. Journal of Animal Ecology, 64, 451–458. 10.2307/5648

[ece36684-bib-0073] Veen, T. , Sheldon, B. C. , Weissing, F. J. , Visser, M. E. , Qvarnström, A. , & Sætre, G. P. (2010). Temporal differences in food abundance promote coexistence between two congeneric passerines. Oecologia, 162(4), 873–884. 10.1007/s00442-009-1544-1 20043178 PMC2841267

[ece36684-bib-0074] Verfaillie, D. , Lafaysse, M. , Déqué, M. , Eckert, N. , Lejeune, Y. , & Morin, S. (2018). Multi‐component ensembles of future meteorological and natural snow conditions for 1500m altitude in the Chartreuse mountain range. Northern French Alps. the Cryosphere, 12(4), 1249–1271. 10.5194/tc-12-1249-2018

[ece36684-bib-0075] Visser, M. E. , & Gienapp, P. (2019). Evolutionary and demographic consequences of phenological mismatches. Nature Ecology & Evolution, 3(6), 879–885. 10.1038/s41559-019-0880-8 31011176 PMC6544530

[ece36684-bib-0076] Visser, M. E. , Holleman, L. J. M. , & Caro, S. P. (2009). Temperature has a causal effect on avian timing of reproduction. Proceedings of the Royal Society B: Biological Sciences, 276(1665), 2323–2331. 10.1098/rspb.2009.0213 PMC2677614 19324731

[ece36684-bib-0077] Visser, M. E. , Holleman, L. J. , & Gienapp, P. (2006). Shifts in caterpillar biomass phenology due to climate change and its impact on the breeding biology of an insectivorous bird. Oecologia, 147(1), 164–172. 10.1098/rspb.2009.0213 16328547

[ece36684-bib-0078] Visser, M. E. , Noordwijk, A. J. V. , Tinbergen, J. M. , & Lessells, C. M., (1998). Warmer springs lead to mistimed reproduction in great tits (parus major). Proceedings of the Royal Society of London. Series B: Biological Sciences, 265(1408), 1867–1870. 10.1098/rspb.1998.0514

[ece36684-bib-0079] Visser, M. E. , Silverin, B. , Lambrechts, M. M. , & Tinbergen, J. M. (2002). No evidence for tree phenology as a cue for the timing of reproduction in tits *Parus spp* . Avian Science, 2(2), 77–86.

[ece36684-bib-0080] Vitasse, Y. , Rebetez, M. , Filippa, G. , Cremonese, E. , Klein, G. , & Rixen, C. (2017). ‘Hearing’ alpine plants growing after snow melt‐out: Ultrasonic snow sensors provide long‐term series of alpine plant phenology. International Journal of Biometeorology, 61(2), 349–361. 10.1007/s00484-016-1216-x 27539023

[ece36684-bib-0081] Vitasse, Y. , Signarbieux, C. , & Fu, Y. H. (2018). Global warming leads to more uniform spring phenology across elevations. Proceedings of the National Academy of Sciences, 115(5), 1004–1008. 10.1073/pnas.1717342115 PMC5798366 29279381

[ece36684-bib-0082] Wipf, S. (2010). Phenology, growth, and fecundity of eight subarctic tundra species in response to snow melt‐out manipulations. Plant Ecology, 207(1), 53–66. 10.1007/s11258-009-9653-9

[ece36684-bib-0083] Wipf, S. , Stoeckli, V. , & Bebi, P. (2009). Winter climate change in alpine tundra: Plant responses to changes in snow depth and snow melt‐out timing. Climatic Change, 94(1), 105–121. 10.1007/s10584-009-9546-x

[ece36684-bib-0084] Yates, K. L. , Bouchet, P. J. , Caley, M. J. , Mengersen, K. , Randin, C. F. , Parnell, S. , … Sequeira, A. M. M. (2018). Outstanding challenges in the transferability of ecological models. Trends in Ecology & Evolution, 33(10), 790–802. 10.1016/j.tree.2018.08.001 30166069

