# Supplementary material for: Best environmental predictors of breeding phenology differ with elevation in a common woodland bird species
Source: Ecol Evol. 2020 Aug 17;10(18):10219–29. doi: 10.1002/ece3.6684 (PMC7520200; doi:10.1002/ece3.6684)
Supplement: Supplementary file 1 — Supinfo [file ECE3-10-10219-s001.docx]

**Supplementary material**

**ESM 1**

Estimation of missing air temperature values of the 1915m station with the station located at 1970m asl.

We estimated the parameters (intercept and slope) of the linear relationship between the two stations (1915m and 1970m) to further estimate missing daily temperature values at 2m for the 1915m station.

Equation: Air temperature(Station 1915m) = Air temperature(Station 1970m)* 1.017 – 0.105

Adjusted r-square = 0.995


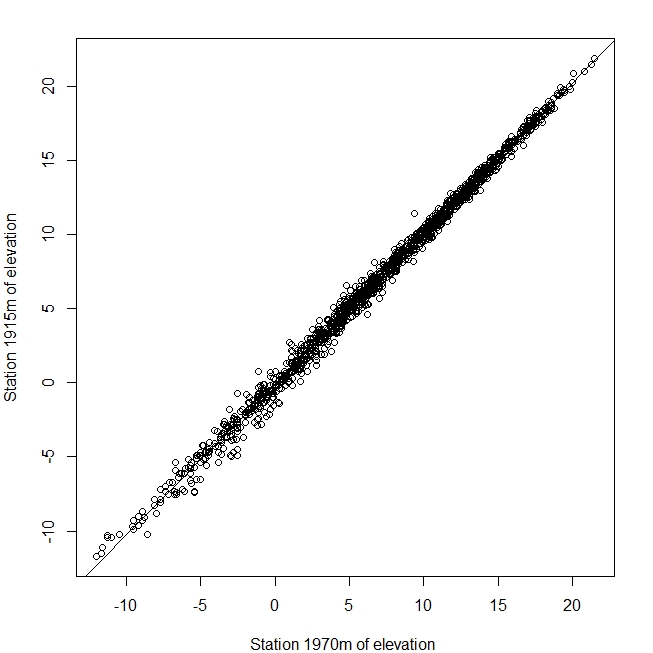
**Figure S1.** Linear relationship between the air temperature (°C) at the two stations (1915m and 1970m asl).

We used the same approach to estimate the average temperature between the 1^st^ of March and 14^th^ of April in 2018 in the high elevation site (NA during this period in 2018 for the station located at 1915m asl).

We estimated the parameters (intercept and slope) of the linear relationship between the stations located at l340m and 1915m asl to further estimate missing daily temperature values at 2m for the high elevation station (1915 asl).

Equation: Air temperature(Station 1915m) = Air temperature(Station 1340m)* 0.99 – 2.69

Adjusted r-square = 0.967

**ESM 2**

**Table S1.** Date of snow melt-out estimated with the sensor at the ground’s surface at low elevation

| Year | Date of Snow melt-out [DOY] |
| --- | --- |
|  | 1340m station |
| 2011 | 63 |
| 2012 | 60 |
| 2013 | 69 |
| 2014 | 70 |
| 2015 | 66 |
| 2016 | 77 |
| 2017 | 52 |
| 2018 | 82 |
| 2019 | 56 |

**Table S2.** Date of snow melt-out estimated with the sensor at the ground’s surface at high elevation

|  | Date of Snow melt-out [DOY] | | Mean date of Snow melt-out [DOY] |
| --- | --- | --- | --- |
| Year | 1915m station | 1970m station |  |
| 2011 | 91 | NA | 91 |
| 2012 | 124 | 125 | 125 |
| 2013 | NA | 153 | 153 |
| 2014 | 100 | 100 | 100 |
| 2015 | 112 | NA | 112 |
| 2016 | 136 | 149 | 143 |
| 2017 | 101 | 100 | 101 |
| 2018 | NA | 140 | 140 |
| 2019 | 141 | 140 | 141 |

**ESM 3**

**Table S1.** Larch budburst date [DOY] in Vallorcine and Montroc

| **Year** | **Vallorcine** | **Montroc** |
| --- | --- | --- |
| 2011 | 95 | 92 |
| 2012 | 100 | 99 |
| 2013 | 114 | 115 |
| 2014 | NA | 98 |
| 2015 | 105.5 | 109 |
| 2016 | 105 | 110 |
| 2017 | 91 | 90 |
| 2018 | 108 | 110 |
| 2019 | 111 | 104 |

**Figure S1.** Larch budburst date by year at the low elevation site near the nestboxes (Vallorcine) and at the site located 5.8km away (Montroc)

*Quantification of inter-individual variation among larches surveyed at low and high elevation*

To quantify inter-individual variation among larches surveyed at low and high elevation sites, we used separate linear mixed models at each elevation (to have independent estimates of both fixed and random effects), with budburst date as response, year as a categorical fixed effect and larch tree as a random effect (budburst date ~ year + (1|larch tree)). Inter-individual and residual variance (Table S2) are much lower than inter-annual variation of budburst date, for which the maximum differences between years equal 25 and 35 days respectively at the low and high elevation sites.

**Table S2.** Inter-individual and residual variance of random effect obtained from the linear mixed models performed at each elevation

|  | Inter-individual variance | Residual variance |
| --- | --- | --- |
| Low elevation site | 5.52 | 4.62 |
| High elevation site | 9.92 | 9.42 |

**ESM 4**

Projection of budburst date

Based on the study of Asse et al. (2020) in Agricultural Forest and Meteorology:

Asse D., Randin C.F., Bonhomme M., Delestrade A., Chuine I., 2019. Process-based models may outcompete correlative models in projecting spring phenology of trees in a future warmer climate

To predict budburst date in 2014 (lack of data) at the high elevation site, we have used a sequential 2-phase model that takes into account the chilling requirements during the endodormancy phase (first phase) and the forcing requirements during the ecodormancy phase (second phase; Chuine et al., 1999).

The endodormancy phase ends at $t_{c}$ when the sum of daily the rate of dormancy release $R_{c}$, reaches the critical value $C$.

$$\sum_{t_{0}}^{t_{c}} R_{c}\left( T_{d} \right)\geq C$$

Budburst occurs at *t*_f_ when the sum of the daily rates of development (*R_f_*) reaches the critical value *F^*^*. From $t_{c}$ to $t_{f}$, forcing units are then accumulated as:

$$\sum_{t_{c}}^{t_{f}} R_{f}\left( T_{d} \right)\geq F$$

In this study the response function to temperature for the endodormancy phase is a threshold function:

$$R_{c}\left( T_{d} \right)=\left\{ \begin{aligned} 1ifT_{d}<T_{b} \\ 0ifT_{d}\geq T_{b} \end{aligned} \right.$$

the response function to temperature (*R_f_*) is the sigmoid function,

$$R_{f}\left( T_{d} \right)=\frac{1}{1+e^{-d_{T}\left( T_{d}-T_{50} \right)}}$$

with $d_{T}$ the steepness of the response and $T_{50}$ the mid-response temperature.

In a first time, for each species model was calibrated using the phenological data and corresponding meteorological data of Phenoclim observation sites equipped with a meteorological station for the 2007-2014 time period. Process-based model was adjusted 20 independent times by minimizing the residual sum of square using the simulated annealing algorithm of Metropolis (Chuine et al., 1998) using the Phenology Modelling Platform software (PMP5; http://www.cefe.cnrs.fr/fr/recherche/ef/forecast/phenology-modelling-platform) (Chuine et al., 2013).

In a second time, we used models calibrated on observation sites with meteorological stations to project the budburst date at each observation site for the 2007-2014 time period.

For more details see Asse et al., 2019 (submitted), where to define the best model different process-based and correlative phenology models have been develop. Several response function and several calibration methods have been tested. Models were calibrated, validated and compared.

Asse, D., Randin, C.F., Bonhomme, M., Delestrade, A. & Chuine, I., 2020. Process-based models outcompete correlative models in projecting spring phenology of trees in a future warmer climate. Agricultural and Forest Meteorology, 285-286, 107931. doi: 10.1016/j.aqrformet.2020.107931

Chuine, I., Cour, P., Rousseau, D.D., 1999. Selecting models to predict the timing of flowering of temperate trees: Implications for tree phenology modelling. Plant, Cell Environ. 22, 1–13. doi:10.1046/j.1365-3040.1999.00395.x

Chuine, I., Cour, P., Rousseau, D.D., 1998. Fitting models predicting dates of flowering of temperate-zone trees using simulated annealing. Plant, Cell Environ. 21, 455–466. doi:10.1046/j.1365-3040.1998.00299.x

Chuine, I., De Cortazar-Atauri, I.G., Kramer, K., Hänninen, H., 2013. Plant development models, in: Phenology: An Integrative Environmental Science. pp. 275–293. doi:10.1007/978-94-007-6925-0_15

**ESM 5**


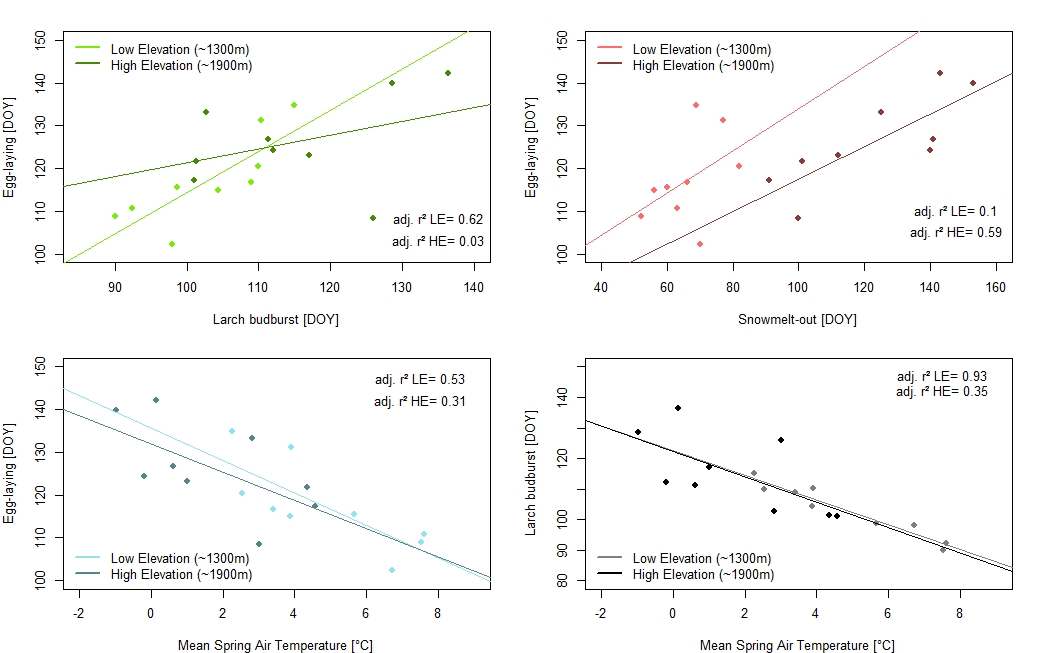


**Figure S1.** Relationships between egg-laying date, air temperature, snow melt-out date and larch budburst date at each elevation

**ESM 6**


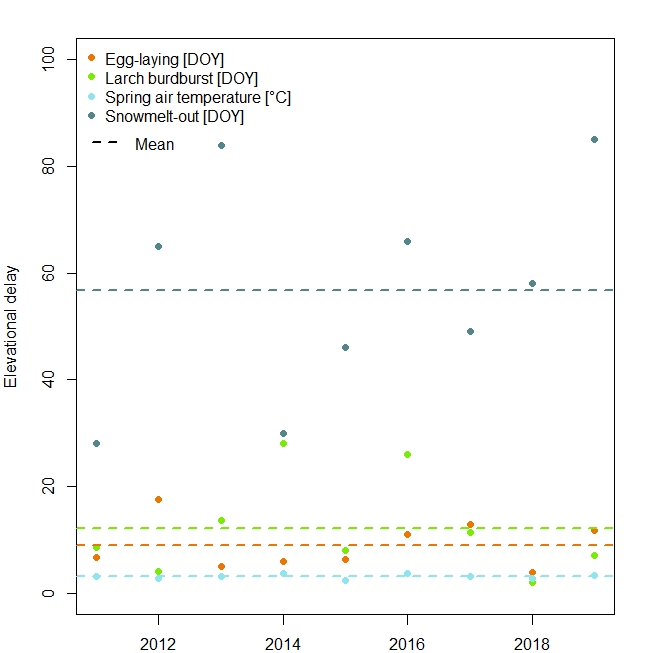


**Figure S1.** Elevational delay between the low and high elevation sites values for each environmental variable
